# Getting to the core: Internal body temperatures help reveal the ecological function and thermal implications of the lions’ mane

**DOI:** 10.1002/ece3.2556

**Published:** 2016-12-20

**Authors:** Paul Trethowan, Andrea Fuller, Anna Haw, Tom Hart, Andrew Markham, Andrew Loveridge, Robyn Hetem, Byron du Preez, David W. Macdonald

**Affiliations:** ^1^ Wildlife Conservation Research UnitThe Recanati‐Kaplan CentreDepartment of ZoologyUniversity of OxfordOxfordUK; ^2^ Brain Function Research GroupSchool of PhysiologyFaculty of Health SciencesUniversity of the WitwatersrandJohannesburgSouth Africa; ^3^ Department of ZoologyUniversity of OxfordOxfordUK; ^4^ Department of Computer SciencesUniversity of OxfordOxfordUK; ^5^ School of Animal, Plant and Environmental SciencesFaculty of ScienceUniversity of the WitwatersrandJohannesburgSouth Africa

**Keywords:** body temperature, ecological function, *Panthera leo*, thermal biology

## Abstract

It has been proposed that there is a thermal cost of the mane to male lions, potentially leading to increased body surface temperatures (*T*_s_), increased sperm abnormalities, and to lower food intake during hot summer months. To test whether a mane imposes thermal costs on males, we measured core body temperature (*T*_b_) continuously for approximately 1 year in 18 free‐living lions. There was no difference in the 24‐hr maximum *T*_b_ of males (*n* = 12) and females (*n* = 6), and males had a 24‐hr mean *T*_b_ that was 0.2 ± 0.1°C lower than females after correcting for seasonal effects. Although feeding on a particular day increased 24‐hr mean and 24‐hr maximum *T*_b_, this phenomenon was true of both male and female lions, and females had higher 24‐hr mean and 24‐hr maximum *T*_b_ than males, on both days when lions did not feed, and on days when lions did feed. Twenty‐four‐hour *T*_b_ was not influenced by mane length or color, and 24‐hr mean *T*_b_ was negatively correlated with mane length. These data contradict the suggestion that there exists a thermal cost to male lions in possessing a long dark mane, but do not preclude the possibility that males compensate for a mane with increased heat loss. The increased insulation caused by a mane does not necessarily have to impair heat loss by males, which in hot environments is primarily through respiratory evaporative cooling, nor does in necessarily lead to increased heat gain, as lions are nocturnal and seek shade during the day. The mane may even act as a heat shield by increasing insulation. However, dominant male lions frequent water points more than twice as often as females, raising the possibility that male lions are increasing water uptake to facilitate increased evaporative cooling. The question of whether male lions with manes compensate for a thermal cost to the mane remains unresolved, but male lions with access to water do not have higher *T*_b_ than females or males with smaller manes.

## 1 Introduction

The most striking feature of the male African lion (*Panthera leo*) is its mane. A thick, dark mane is thought to have advantages for males that possess it, although hypotheses vary as to which benefits are the most important. Charles Darwin and others proposed that the lion's mane may provide a signal of fighting prowess to other males, helping to avoid unnecessary conflicts, and protecting the neck when conflicts do occur (Darwin, 1871; Schaller, 1972). Alternatively, the lion's mane may simply be ornamental and mate choice may be the primary evolutionary force, as suggested by the finding that females preferentially move toward model male lions with darker manes (West & Packer, 2002). Indeed, by selecting males with darker manes, female lions are indirectly selecting more mature, healthy males, with higher testosterone levels, which may be able to better provide for and protect their prides (West & Packer, 2002).

Sexually selected traits have obvious advantages, but if they can be realized without a cost, all individuals in a population may develop those traits to a similar extent, neutralizing the usefulness of the signal. The necessity of this cost is known as the handicap principle (Zahavi, 1977). Evidence suggests that the cost of a lion's mane may be a thermal one. As early as 1908, Frederick Selous, the famous British hunter‐naturalist, proposed that the primary factor influencing the size of a lion's mane was ambient temperature (Selous, 1908). More recently, West and Packer (2002) found that body surface temperatures (*T*_s_), measured by infrared thermography shortly after sunset, were higher for male lions with manes than for females and that those males with darker manes tended to have higher *T*_s_ than males with lighter or no manes. The high *T*_s_ of dark‐maned lions may represent a cost, particularly during the hot months when the high *T*_s_ was linked to sperm abnormalities and reduced food intake (West & Packer, 2002). Indeed, male lions in warmer climates generally have smaller manes, as demonstrated for captive lions across latitudes in North America (Patterson, Kays, Kasiki, & Sebestyen, 2006), and the small‐maned Asian lion in India (Barnett, Yamaguchi, Shapiro, & Nijman, 2007).

The existing experimental evidence to suggest that males with long dark manes may incur a thermal cost is therefore based on a single study of *T*_s_ measurements. However, physiological performance is influenced by core body temperature (*T*_b_), and not *T*_s_. External factors such as air temperature, as well as peripheral vasomotor control, influence *T*_s_ (Porter & Gates, 1969), and higher *T*_s_ of one individual than of another, at a particular time of day, do not necessarily represent a higher *T*_b_. Indeed, if environmental temperature is lower than *T*_b_, as is likely after sunset when initial infrared thermography measurements were made, then a higher *T*_s_ may reflect greater peripheral vasodilation to dissipate heat and reduce *T*_b_. Further, a higher *T*_s_ on the flanks of male lions may reflect increased vasomotor control to direct peripheral blood flow away from the areas covered by the mane and toward areas where heat can be more easily dissipated, such as the loins and flanks. Thus, *T*_b_ and *T*_s_ are not well correlated and it is important to measure *T*_b_ when attempting to assess the thermal state of an animal.

To further investigate whether there is a thermal cost of the lion's mane, we measured *T*_b_ for approximately 1 year in 18 free‐living lions (six females, 12 males), in a hot semi‐arid environment where lions develop prominent manes. We compared the *T*_b_ (24‐hr mean and maximum) of male and female lions across different seasons, and *T*_b_ of lions with short and long manes or dark and light manes. We also compared lion *T*_b_ on days when the animals fed and did not feed, as it has been reported that males reduce food intake due to the interaction between the specific dynamic action of digestion and the thermal burden of the mane (West & Packer, 2002). If there is a thermal cost of the mane, resulting in increased heat gain or reduced heat dissipation, it is possible that male lions may maintain a *T*_b_ no different to that of females, as a result of increased evaporative heat loss. To investigate this, we also measured the frequency and duration of water intake in males and females, as increased evaporative heat loss would likely be associated with greater water intake.

## 2 Materials and Methods

### 2.1 Ethical note

All animal procedures carried out in this study received appropriate approval from the University of Oxford Animal Welfare and Ethical Review Board and the University Veterinary Services Department. Procedures were also approved by the Animal Ethics Screening Committee of the University of the Witwatersrand (clearance certificate no. 2013/54/04). A veterinarian carried out the surgical procedures and project staff that handled animals during captures were qualified to do so by attendance at Zimbabwe's Physical and Chemical Capture of Wild Animals Course and held valid drugs licenses (Dangerous Drugs License No. 2014/16). Permission was granted from the landowner and conservancy management, following the ASAB/ABS recommendations for the Use of Animals in Research, for the capture and handling of the study animals.

### 2.2 Study site

This study took place at the Bubye Valley Conservancy (BVC), a privately owned wildlife conservancy located in southern Zimbabwe. The BVC is approximately 3,700 km^2^ and is located in the lowveld region of Zimbabwe between latitudes 21.2° and 21.8° south, and longitudes 29.8° and 30.5° east, at an elevation of 500–600 m above sea level (see du Preez, Loveridge, and Macdonald (2014)). The lowveld region is in one of the hottest and driest areas in Zimbabwe with summer temperatures regularly exceeding 40°C, and mean annual rainfall between 2007 and 2012 was 351 ± 76 mm (du Preez, 2014). Bubye Valley Conservancy is a fenced wildlife conservancy with a full complement of indigenous megafauna (du Preez, 2014) and contains one of Zimbabwe's largest lion populations, recently estimated to be between 330 and 550 individuals (2016 population survey, data not shown). The BVC is a mesotrophic ecosystem characterized by mopane (*Colophospermum mopane*) woodland and scrubland.

### 2.3 Weather data

Weather data were measured using a portable weather station (HOBO^®^ Weather Station Data Logger—H21‐001, Onset Computer Corporation, Massachusetts, USA) erected in an open area at the study site and programmed to log measurements at five‐minute intervals. Weather parameters measured included dry bulb air temperature, black globe temperature, relative humidity, solar radiation, wind speed, and wind direction. We used a standard rain gauge to measure daily precipitation. Seasonal weather conditions are summarized in Table S1.

### 2.4 Bio‐loggers

To monitor animals and track their movements, all study lions were fitted with GPS satellite radio‐telemetry collars (African Wildlife Tracking, Pretoria, South Africa), programmed to record geographical locations once per hour from 17:00 to 07:00 local time and at 12:00 and 14:00.

Surgically implanted miniature temperature‐sensitive bio‐loggers (DST centi loggers, Star Oddi, Gardabaer, Iceland) covered in inert surgical wax (Sasolwax 1276, Sasol Wax GmbH, Worthdamm 13‐27, D‐20457, Hamburg, Germany) were used to measure *T*_b_ at 5‐min intervals. The miniature temperature bio‐loggers (hereafter referred to as temperature bio‐loggers) had a measurement resolution of 0.03°C and a range of 5–45°C. We used a high accuracy thermometer (Quat 100, Heraeus, Hanau, Germany) to calibrate temperature bio‐loggers in an insulated water bath at temperatures ranging from 30 to 42°C. Temperature bio‐loggers were calibrated at both the start and end of the study; there was no evidence of calibration drift over time, and bio‐loggers were accurate to 0.1°C postcalibration. Data were retrieved by recovering the temperature bio‐loggers and downloading the stored temperature data.

We recorded lion activity and acoustic data using custom‐built accelerometer bio‐loggers (Biotrack/University of Oxford, accelerometer data recorded at 16 Hz, 3 dimensions) and acoustic accelerometer bio‐loggers (Biotrack/University of Oxford, acoustic data recorded at 8 bit, 16 kHz mono, and accelerometer data at 16 Hz, 3 dimensions).

It was not possible to continuously observe multiple lions simultaneously over several months. We therefore used acoustic bio‐loggers to obtain a continuous audible record of each lions’ activities. The acoustic accelerometer and accelerometer bio‐logger components were covered in dental acrylic for protection, and the bio‐loggers were mounted on curved copper plates and bolted to GPS collars. Both acoustic accelerometer and accelerometer bio‐loggers were identical in shape and size, approximately 50 × 20 × 30 mm, and weighed <150 g. Acoustic accelerometer bio‐loggers recorded audio and accelerometer data simultaneously from the time the bio‐logger was turned on (at the time of capture) until the device terminated due to battery failure (approximately 1 week later). Accelerometer bio‐loggers recorded accelerometer data from the time of capture until battery failure (approximately 3–6 months later). Audio and accelerometer data were stored on a standard micro‐SD card, and the data were obtained by recovering the bio‐logger and extracting the micro‐SD card.

### 2.5 Study animals

Between February 2010 and February 2015, 31 lions (14 female, 17 male) in BVC were captured and fitted with GPS satellite radio‐telemetry collars (African Wildlife Tracking, Pretoria, South Africa) and monitored regularly as part of a long‐term research project. The GPS satellite collars were regularly used to find and observe the study animals (weekly–monthly). Field observations noted behavior, body condition, apparent territorial status, and other lions present with the collared study animal.

In January 2014, we re‐captured and fitted 18 of these study lions (six female, 12 male) with temperature bio‐loggers. All females were mature adults that had previously suckled cubs and ranged in age from 5 to 9 years. Males ranged in age from 3.5 to 10 years old. We simultaneously fitted accelerometer bio‐loggers to 10 of the study animals (three female, seven male) that received temperature loggers. During the study, in November 2014, we re‐captured eight of the study lions (three female, five male), removed their accelerometer bio‐loggers if they had been previously fitted with one, and fitted the lions with acoustic accelerometer bio‐loggers. A summary of the individuals used in this study and the various bio‐loggers that were fitted is shown in Table S2.

We recovered 840 days of accelerometer data (range: 20–146 days per lion, mean: 84 ± 48 days per lion), from the 10 study animals that were fitted with both temperature bio‐loggers and accelerometer bio‐loggers. We recovered a total of 60 days of simultaneous audio and accelerometer data from the acoustic accelerometer bio‐loggers (mean: 7.5 ± 2.4 days per lion).

### 2.6 Capture procedure and mane measurements

Lions were approached by vehicle and darted from a distance of 10 to 35 m, with 1 cc darts (Type P, ¾ “ needle with gel collar, Pneudart, Williamsport, Pennsylvania, USA), loaded with a tiletamine:zolazepam combination (Zoletil, 75–100 mg, 250 mg/ml, Virbac RSA (Pty) Ltd., Halfway House, South Africa) and medetomidine (5 mg, 50 mg/ml, Kyron Laboratories, Johannesburg, South Africa). Darts were delivered by a CO_2_‐pressurized dartgun (Dan‐Inject, Børkop, Denmark). Once the animal was immobilized (12–15 min later), it was approached carefully and a blindfold was placed over the eyes. The front legs were secured with a rope, and earplugs inserted to dampen auditory stimuli.

**Figure 1:**
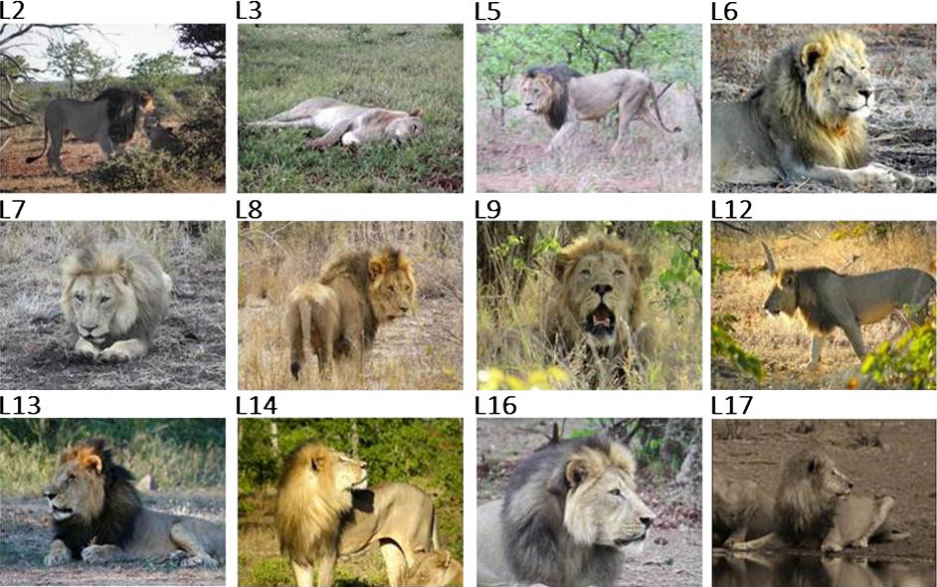
Photographs of all 12 male lions and mane color classification, included in this study. L, lion ID. Mane shade classification: light = L3, L7, 17; medium = L6, L8, L9, L12, L14; dark = L2, L5, L13, L16

While lions were immobilized for bio‐logger fitting, we took measurements of mane length (mm) for male lions. Mane hairs were pulled straight and measured with a standard ruler at the crest of skull, crest of shoulder, point of jaw, and center of chest. The various mane length measurements were then summed to give a total length score that was used as a proxy for mane length. We also classified each mane according to the predominant mane color as dark, medium, or light, based on visual assessment of the mane (Figure 1).

After bio‐loggers had been fitted, the effects of medetomidine were reversed with atipamezole (Antisedan 5 mg/ml, Pfizer Animal Health, Johannesburg, South Africa) at a dose of 2.5–5 times the total medetomidine dose. We administered half the atipamezole intramuscularly and the other half intravenously. Lions took between 15 and 90 min from the time of antidote administration to recover sufficiently from the drug effects in order to be able to walk, but were monitored for a minimum of 3 hr postcapture until normal activity was resumed, as determined by veterinary staff. Animals were monitored opportunistically for 10 days postsurgery and intermittently thereafter. No problems were observed with recovery, and all lions returned to their social groups within a few hours of the surgical procedure.

### 2.7 Surgical procedure

Using sterile surgical procedures, temperature bio‐loggers were implanted between the parietal peritoneum and the transversus abdominis muscle via an incision in the paralumbar fossa. The loggers were dry‐sterilized in formaldehyde vapor before implantation. After administering a local anesthetic (5 ml 2% lignocaine hydrochloride, Bayer Animal Health (Pty) Ltd. Isando, South Africa) subcutaneously, the surgical site was shaved and sterilized with 5% chlorhexidine gluconate (Hibitane, Astra Zeneca, Johannesburg, South Africa) in 100% ethanol. Temperature loggers were tethered to the abdominal muscle wall with nylon (0 USP, Scimitar Surgical Sutures, Gabler Medical (Pty) Ltd., Cape Town, South Africa) to ensure their original position was maintained. Once the loggers were implanted, the incision was closed with absorbable suture material (Viamac, Scimitar Surgical Sutures, Glaber Medical (Pty) Ltd., Cape Town, South Africa) and then treated with a topical antiseptic spray (Necrospray, Centaur Labs, Johannesburg, South Africa) and covered with tick grease (chlorfenvinphos 0.3%, Swavet Tick Grease, Swavet RSA (Pty) Ltd.). The lions received a long‐acting antibiotic (6,000 IU/kg of intramuscular procaine benzylpenicillin, Duplocillin, Intervet, South Africa) and a nonsteroidal anti‐inflammatory analgesic (0.2 mg kg^−1^ of subcutaneous meloxicam, Metacam, Boehringer Ingelheim, Johannesburg, South Africa).

### 2.8 Data analysis

#### 2.8.1 Determining feeding

Accelerometer data were classified to infer feeding or nonfeeding, using machine learning techniques trained with labeled data from acoustic accelerometer bio‐loggers. Raw accelerometer data (collected at 16 Hz) from both accelerometer and acoustic accelerometer bio‐loggers were summarized into five‐minute periods associated with 12 descriptive feature parameters (mean acceleration and mean variance of acceleration in the X, Y, and Z dimensions; mean and variance of overall dynamic body action (ODBA); and the mean and variance of the pitch and roll of the bio‐logger). We constructed a large dataset of labeled accelerometer data from the acoustic accelerometer bio‐loggers by listening to recordings of all the audio data and labeling the simultaneously collected accelerometer data, as either feeding or nonfeeding behavior. Feeding behavior was determined by growls, chewing, and bone crunching sounds (Audio files S1 and S2). From the audio dataset, we labeled 47 hr of recorded feeding behavior, and 769 hr of nonfeeding behavior. The labeled accelerometer data were reorganized into a random order and then classified using a random forest method (Breiman, 2001), with 1,000 trees and four variables per split. The classification model achieved an out‐of‐bag (OOB) estimated error rate of 1.7%. Feeding was misclassified as nonfeeding behavior at an error rate of 22.2%, and nonfeeding behavior was classified as feeding at a class error rate of 0.4%.

After examining the predicted classification against the known behavior (from simultaneous audio recordings), we discovered that the majority of the false‐positive classification errors (nonfeeding behavior classified as feeding) were characterized by single isolated feeding events (i.e., a five‐minute feeding event flanked by nonfeeding events). We then added a second classification layer that removed single isolated feeding events and compared the predicted behavior against the entire labeled dataset. Consequently, we generally underestimated the amount of time spent feeding during a feeding episode (i.e., a meal) because the error rate for feeding classified as nonfeeding was 22.2% and single isolated feeding events were removed. However, all feeding episodes (meals, 44) in the 60 days of recorded lion behavior were detected (Table 1), and no false‐feeding events were inferred. We then used the random forest model, and second classification layer, to classify the accelerometer data obtained from accelerometer bio‐loggers (3–6 months of continuous data per lion).

**Table 1:** Summary of total audio data collected per lion (days), clear audio data remaining after removing low‐quality recordings (days), and the number of feeding events and drinking events recorded

ID	Sex	Total audio	Clear audio	Feed	Drink
Lion 7	Male	6.8	0	3	0
Lion 8	Male	10.9	10.9	5	11
Lion 13	Male	11	8.8	5	16
Lion 14	Male	8.8	5.2	5	5
Lion 16	Male	3.2	3.2	2	4
Lioness 4	Female	7.1	4.4	7	4
Lioness 7	Female	8.9	8.8	8	13
Lioness 14	Female	8.9	8.4	9	11

The classified data from the accelerometer bio‐loggers were used to determine whether or not lions with temperature loggers fed on a particular day (binary variable referred to as “feed day”), and if lions did feed on a particular day, we used the time spent feeding (the number of five‐minute periods classified as feeding, divided by 12 to convert to hours) as a proxy for meal size.

#### 2.8.2 Determining water intake

To determine water intake during drinking events, we listened to the audio data recovered from the acoustic accelerometer bio‐loggers for the sounds of lions drinking (characterized by consistent lapping sounds lasting between two and 14 min, interspersed with a “swallow” sound approximately every 30 s (Audio file S3) and labeled them using Audacity 2.1.1. We measured the time spent drinking (accurate to about 1 s) and the “lap rate” (“laps per second”) for individual drinking events, which were accurate to about 1 s. Occasionally lions drank for several minutes, paused for a break (lasting several seconds to several minutes), and then continued drinking. We considered these instances to be part of the same drinking event and summed the individual drinking bouts to determine the total time spent drinking. “Lap rate” was determined by counting the number of audible “laps” made per minute of continuous drinking for each lion. One‐minute periods were taken from at least three separate drinking events for each of the seven lions for which we had clear audio data. Audio data that were not of sufficient quality were excluded from analysis. A total of 49.7 days of clear audio data were obtained from seven study lions (three female, four male, all resident individuals). These data are summarized in Table 1. To estimate the frequency of drinking, we used GPS data from the lions’ collars to compare the frequency of visits to known water points. Only collar data collected between the beginning of June and the end of August of each year between 2010 and 2014 were included in the analysis because this period represents the late dry season at the study site, when surface water is only available at known locations. We included as many collared lions as possible from the long‐term study that had at least 80 days of continuous collar data available for the June–August period of each year (females 1,784 days of collar data from 13 lionesses, males 1,710 days of collar data from 13 lions; Table S3). All available surface water was accurately marked using a handheld GPS (Garmin, eTrex 10, USA). Analysis was carried out in ARCGIS 10.1. Surface water point locations were added as a GIS layer, and a 75‐m buffer (the approximate area of a water point and surrounding piosphere) was added around each point. Visits to water points were inferred from lion movement trajectories that intersected with water point locations. For each lion, we calculated the frequency of visits to water per day by dividing the number of visits to water, by the number of days for which collar data were available.

### 2.9 Statistical analyses

All statistical analyses were carried out using the software program R (R Core Team, 2015). We calculated 24‐hr summary statistics of mean, minimum, maximum, and amplitude (difference between 24‐hr maximum and minimum) *T*_b_ for each 24‐hr period for each individual. As lions are nocturnal, the 24‐hr period was taken from 12:00 (noon local time) to 11:59 the next day, to capture activity bouts in single time periods. Female *T*_b_ during pregnancy (three females experienced pregnancy) was excluded because we observed different thermal patterns of *T*_b_ in pregnant females (data not shown). Female *T*_b_ during lactation was not different to that in nonlactating females (data not shown) and was thus included in the analysis. When we analyzed the effects of sex and mane characteristics on *T*_b_, we summarized *T*_b_ parameters for each individual in each meteorological season from the 24‐hr summary periods. When assessing the relationship between feeding and *T*_b_, we used the 24‐hr summaries for each individual without summarizing by season. We also calculated the mean absolute maximum *T*_b_ of males and females.

The absolute recorded maximum *T*_b_ of males and females was compared using Student's *t* test. We used the package *nlme* (Pinheiro, Bates, Debroy, & Sarkar, 2011) to perform a linear mixed‐effects analysis of various fixed effects on *T*_b_. The best performing model was then selected based on Akaike Information Criterion corrected for small sample size (AICc) using the R package *MuMin* (Barton, 2015). We did not use model averaging which generates misleading unreliable estimates where predictor variables are correlated (Cade, 2015).

We conducted a linear mixed‐effects analysis of the relationship between *T*_b_, and season and sex. For both seasonal 24‐hr maximum *T*_b_ and seasonal 24‐hr mean *T*_b_, we tested multiple a‐priori candidate models (Table S4) with season and sex as fixed effects and a possible interaction between them. Individuals were included as a random intercept in all models. We also carried out a linear mixed‐effects analysis to investigate the relationship between “feed day” (whether or not the lion fed on a particular day), sex, black globe temperature, and *T*_b_ (Table S5). We included 24‐hr maximum black globe temperature, sex, and the binary categorical variable feed day, as fixed effects. As dependant variables, we used 24‐hr maximum *T*_b_ in the first analysis and 24‐hr mean *T*_b_ in subsequent analysis. Individuals were included as random intercepts in all models. Males and females might have different *T*_b_ responses to feeding or black globe temperature, so we included interaction effects between these terms, and because there was likely to be temporal auto‐correlation, we included an autoregressive correlation structure of order one.

We used a linear mixed‐effects analysis to determine the relationship between mane size and color on 24‐hr mean and 24‐hr maximum *T*_b_. Our dependent variables were seasonal 24‐hr maximum *T*_b_ in the first analysis and seasonal 24‐hr mean *T*_b_, in subsequent analysis. Season, mane length, and mane color were included as fixed effects, and individuals were included as random effects (Table S6).

The duration, lap rate, and total number of laps, during male and female drinking events, were compared using linear models, with sex as the explanatory variable. Individuals were included as a random effect because multiple measurements were taken for each individual. A *p*‐value was obtained with a Satterthwaite approximation (Kuznetsova, Brockhoff, & Christensen, 2015). We used the same method to compare the frequency of visits to water points where the same individuals were measured in different years; a linear mixed‐effects model with the individual included as a random effect and sex and territorial status as fixed effects. An interaction term was included between the effects of sex and territorial status to test for the possibility that the effect of sex depended on territorial status.

## 3 Results

### 3.1 Weather

As is typical of the study area, weather conditions were generally hot and dry. Dry bulb air temperature ranged from an absolute minimum of −0.5°C recorded in winter to a maximum of 46.1°C recorded in spring, and black globe temperature ranged from −1.6°C to 61.9°C, in the same respective seasons. Lions were routinely exposed to high temperatures. Maximum dry bulb air temperature exceeded 38.0°C on 39 days during the study and maximum black globe temperature exceeded 38.0°C on 276 days during the study. A complete summary of the weather parameters per season is given in Table S1.

### 3.2 Effect of sex and season on *T*_b_

Analysis of 24‐hr maximum *T*_b_ revealed that the best performing model included an effect of season (*F*_3,43_ = 4.4, *p* = .009) but not sex, a closer inspection of the second best performing model revealed that sex did not have a significant effect on 24‐hr maximum *T*_b_ (*F*_1,16_ = 2.4, *p* = .1)). However, analysis of 24‐hr mean *T*_b_ included a relationship between both sex (*F*_1,16_ = 6.6, *p* = .02) and season (*F*_3,43_ = 11.5, *p* < .001) on 24‐hr mean *T*_b_, which was an average of 0.2 ± 0.1°C lower in males than in females across seasons. There was no difference (*t*(16) = 1.0, *p* = .4) in the absolute maximum recorded *T*_b_ of males (41.0 ± 0.5°C) and females (40.8 ± 0.3°C). Mean seasonal parameters of *T*_b_ for males and females are summarized in Table 2.

**Table 2:** Mean and standard deviation of *T*_b_ parameters for males (M) and females (F) in each season

*T*_b_ (°C)	Season							
	Dec–Feb “Summer”		Mar–May “Autumn”		Jun–Aug “Winter”		Sep–Nov “Spring”	
	M (*n* = 12)	F (*n* = 6)	M (*n* = 12)	F (*n* = 6)	M (*n* = 10)	F (*n* = 5)	M (*n* = 8)	F (*n* = 5)
Mean	37.7 ± 0.1	37.8 ± 0.1	37.5 ± 0.2	37.7 ± 0.1	37.3 ± 0.3	37.5 ± 0.5	37.5 ± 0.2	37.7 ± 0.1
Minimum	36.7 ± 0.2	36.7 ± 0.3	36.3 ± 0.2	36.8 ± 0.2	35.9 ± 0.4	36.3 ± 0.5	36.0 ± 0.3	36.4 ± 0.3
Maximum	38.8 ± 0.1	39.0 ± 0.1	38.7 ± 0.2	38.8 ± 0.1	38.6 ± 0.4	38.6 ± 0.6	38.8 ± 0.2	39.0 ± 0.1
Amplitude	2.1 ± 0.2	2.3 ± 0.3	2.5 ± 0.3	2.1 ± 0.2	2.7 ± 0.6	2.3 ± 0.3	2.8 ± 0.5	2.6 ± 0.3

*N* denotes the number of individuals.

### 3.3 Body temperature on days when lions did and did not feed

The best performing model predicting 24‐hr maximum *T*_b_ included effects of both feed day (*F*_1,824_ = 18.0, *p* < .001) and sex (*F*_1,8_ = 10.0, *p* = .01), but no effect of 24‐hr maximum globe temperature or interaction effects. On days when lions fed, 24‐hr maximum *T*_b_ was an average of 0.2 ± 0.1°C higher than on days when they did not feed. After correcting for feeding, 24‐hr maximum *T*_b_ of males was an average of 0.3 ± 0.1°C lower than females.

Feed day (*F*_1,822_ = 43.0, *p* < .001), sex (*F*_1,8_ = 6.5, *p* = .03), and 24‐hr mean black globe temperature (*F*_1,822_ = 19.4, *p* < .001) had an effect on 24‐hr mean *T*_b_. On days when lions fed, 24‐hr mean *T*_b_ was an average of 0.2 ± 0.0°C higher than on days when they did not feed (Table 3). After correcting for the effects of feeding and black globe temperature, males had a 24‐hr mean *T*_b_ that was 0.8 ± 0.2°C lower than females. There was an interaction between feed day and sex (*F*_1,822_ = 8.1, *p* = .005), with 24‐hr mean *T*_b_ increasing more in males than females on days when they fed. However, this difference was small (female *T*_b_ increased by 0.17°C compared to male *T*_b_ by 0.18°C on feed days) and likely to be of no biological importance. Twenty‐four‐hour mean and 24‐hr maximum *T*_b_ on days when lions did and did not feed is summarized for males and females in Table 3.

**Table 3:** The 24‐hr maximum and 24‐hr mean *T*_b_ for males and females on days with and without feeding

	24‐hr mean *T*_b_ (°C)		24‐hr maximum *T*_b_ (°C)	
	No feed	Feed	No feed	Feed
*Sex*				
Male (*n* = 10)	37.4 ± 0.1	37.8 ± 0.2	38.3 ± 0.2	38.8 ± 0.2
Female (*n* = 3)	37.6 ± 0.2	37.9 ± 0.2	38.5 ± 0.4	39.1 ± 0.3

Data are shown as the mean with standard deviation between individuals. Number of individuals indicated in parenthesis.

### 3.4 Mane length and color

**Figure 2:**
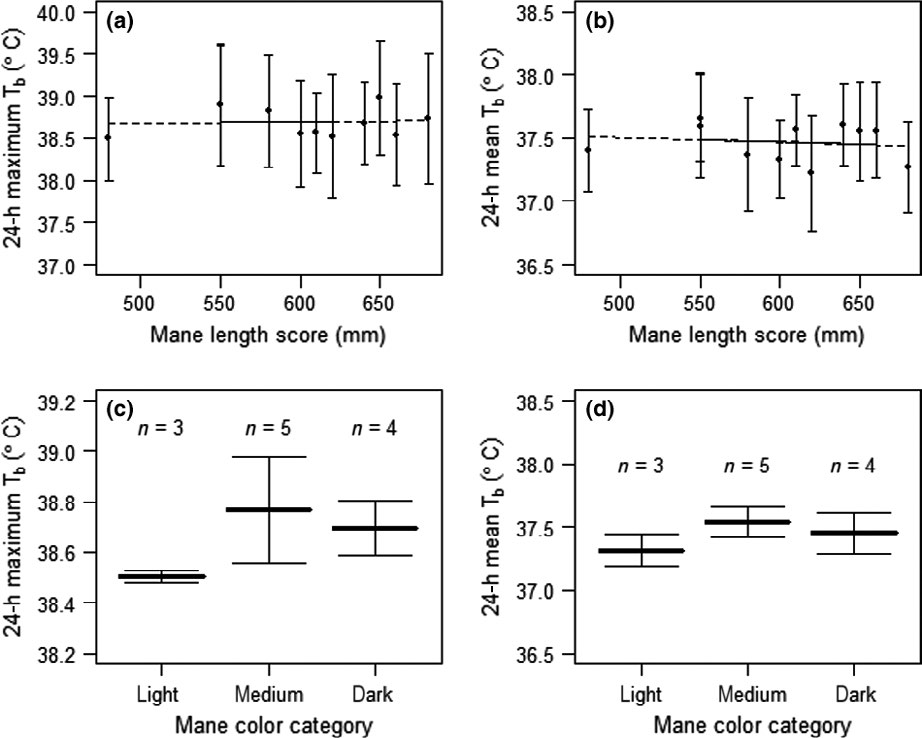
The 24‐hr maximum and mean *T*_b_ of male lions by mane color class and length score (a) shows the 24‐hr maximum *T*_b_ (°C) for each male lion and (b) the 24‐hr mean *T*_b_ for each male lion, against mane length score (mm), (c) shows the mean 24‐hr maximum *T*_b_ between individuals and (d) the mean 24‐hr mean *T*_b_ between individuals, against mane color category from light to dark. For mane color, *n* indicates the number of individuals; the mean is calculated between individuals. Error bars indicated one standard deviation from the mean. *T*_b_ = core; body temperature

As shown by our study animals, male lions on BVC develop prominent manes and display a range of mane lengths and colors (Table 4 and Figure 1). The best performing model predicting 24‐hr maximum *T*_b_ did not include an effect of mane length or mane color (Figure 2a,c). However, there was a relationship between both mane length (*F*_1,7_ = 5.8, *p* = .05; Figure 2b) and mane color (*F*_2,7_ = 6.4, *p* < .03; Figure 2d) on 24‐hr mean *T*_b_. After correcting for season, there was a 0.1°C decrease in 24‐hr mean *T*_b_ for each 100 mm increase in total mane length (Figure 2b). The relationship between 24‐hr mean *T*_b_ and mane color was not as linear as might have been predicted; medium‐colored manes were associated with the highest 24‐hr mean *T*_b_ (Figure 2d). Light‐colored manes were associated with a 24‐hr mean *T*_b_ that was about 0.3 ± 0.1°C lower than medium‐colored manes, and dark manes were associated with a 24‐hr mean *T*_b_ that was 0.1 ± 0.1°C lower than medium‐colored manes.

**Table 4:** Lion mane measurements (mm), length score, and color class

ID	Mane measurements					Color class
	Skull	Shoulder	Jaw	Chest	Length score	
Lion 2	100	200	100	180	580	Dark
Lion 3	140	50	90	200	480	Light
Lion 5	190	190	90	210	680	Dark
Lion 6	120	160	100	220	600	Medium
Lion 7	160	170	90	200	620	Light
Lion 8	190	190	80	200	660	Medium
Lion 9	60	180	90	230	550	Medium
Lion 12	80	160	80	230	550	Medium
Lion 13	150	150	100	240	640	Dark
Lion 14	100	200	100	250	650	Medium
Lion 16	180	130	90	220	610	Dark
Lion 17^a^	–	–	–	–	–	Light

a No reliable mane measurement was recorded for Lion 17.

### 3.5 Frequency and duration of drinking

**Figure 3:**
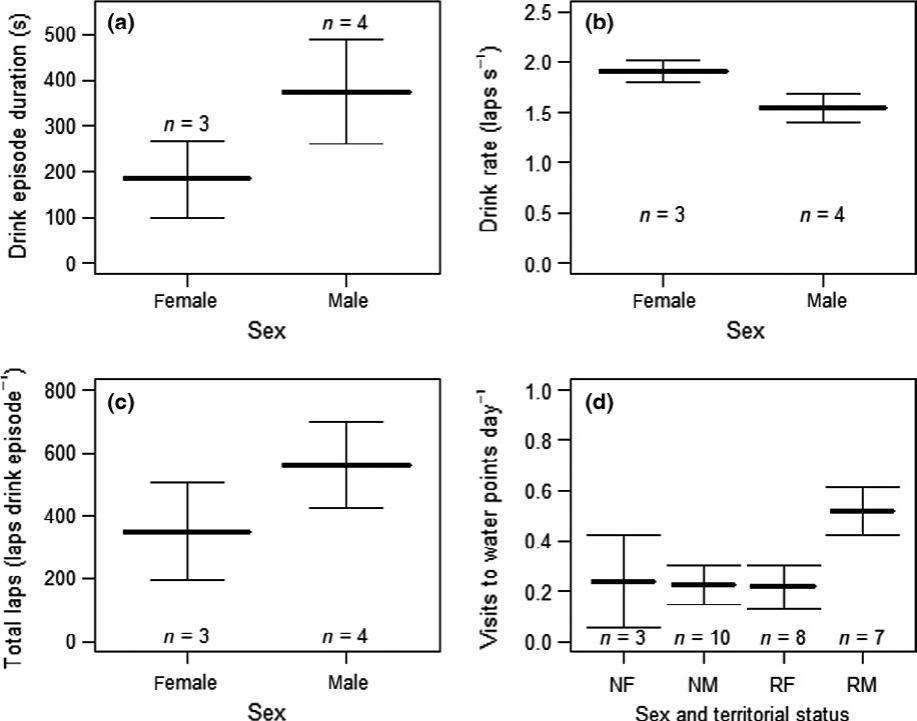
Drinking behavior and frequency of visits to water points in lions. (a) The mean duration of male and female drinking episodes (seconds). (b) The mean lap rate (laps per second) during drinking for males and females. (c) Mean number of total laps for males and females during drinking episodes. (d) Mean number of visits to water points per day by sex and territorial status (NF, nomadic female; NM, nomadic male; RF, resident female; RM, resident male). Means calculated between individuals. Error bars show standard deviation from the mean. *N* shows the number of individuals

Male lions drank for longer (374 ± 113 s) than females (184 ± 84 s) during drinking episodes recorded on the acoustic accelerometer data loggers (*F*_1,5_ = 5.9, *p* = .06; Figure 3a). Sex affected lap rate (*F*_1,5_ = 22.1, *p* = .005), with male lap rates (1.5 ± 0.06 laps s^−1^) being about 0.4 laps s^−1^ slower than female lap rates (1.9 ± 0.04 laps s^−1^; Figure 3b). Consequently there was no significant difference in the total number of laps per drinking episodes between males and females (*F*_1,5_ = 2.3, *p* = .2), although the mean number of laps was higher for males than females (Figure 3c). There was strong evidence that the effect of sex, on the frequency of visits to water, depended on territorial status (*F*_1,11_ = 19.7, *p* < .001). Resident male lions visited water points more than twice as often as females or nomadic males (Figure 3d), and there was no difference in the frequency of visits to water points between nomadic males and females (*F*_1,24_ = 0.3, *p* = .8), or dominant females and nomadic males and females (*F*_1,11_ = 0.4, *p* < .7).

## 4 Discussion

To determine whether there may be a thermal cost to male lions in possessing a mane, we investigated the thermal biology of the lion by measuring *T*_b_ continuously for approximately 1 year in 18 free‐living lions, the largest cohort of free‐living large mammals for which *T*_b_ has been recorded over an extended period of time. Our study was carried out in a hot semi‐arid environment where daytime temperatures frequently exceed *T*_b_, and where lions are known to develop prominent manes. Despite the hot conditions and the large, dark manes of some of the study animals, male lions had a 24‐hr mean *T*_b_ that was about 0.2°C lower than that in females, and there was no difference between male and female 24‐hr maximum *T*_b_. In addition, between males, both mane color and length did not influence 24‐hr maximum *T*_b_, and 24‐hr mean *T*_b_ was negatively correlated with mane length. Thus, our results, derived from the first measurements of *T*_b_ in free‐living lions, do not suggest that the presence and form of the mane are associated with a thermal cost.

Lions are capable of making efficient use of moisture derived from their prey (Eloff, 1973; Owens & Owens, 1984), so feeding also will provide a water source to support evaporative cooling and regulation of *T*_b_ in hot conditions. Because male lions did not have higher *T*_b_ than female lions, it seems unlikely that male lions would need to reduce their feed intake during hot periods to avoid increased heat loads, as inferred by the observation that male lions had smaller bellies than did females in hot months (West & Packer, 2002). Postprandial‐specific dynamic action, the cost of digesting food and the associated increase in metabolic activity, is well documented in the literature and known to increase *T*_b_ in some species (Secor, 2009). In cheetah, however, the only other free‐living felid in which *T*_b_ has been measured, an increase in *T*_b_ after hunting was associated with stress hyperthermia and not feeding (Hetem et al., 2013). Our data do not support the idea that male lions need to reduce food intake during hot conditions. On days when lions fed, both sexes had higher 24‐hr mean and 24‐hr maximum *T*_b_ than on days during which they did not feed and the increase in temperature with feeding was lower for males than for females. We did not attempt to determine whether elevated *T*_b_ was caused by activity (hunting), specific dynamic action, or stress hyperthermia (as likely in the case of cheetah; [Hetem et al., 2013]), as we were interested in whether males needed to reduce food intake as a consequence of higher *T*_b_. We believe it is unlikely, therefore, that smaller belly sizes observed in male lions in hot months reflect reduced feeding associated with regulating *T*_b_. Less distended bellies in males in hot months could result from a variety of factors. For example, in hot months, lions may require less food intake and dominant males with territories to defend (and their associated large manes) may devote more time to territorial defense than feeding.

Our results, that male lions with long dark manes did not have higher *T*_b_ than females or males with smaller manes, can be explained in one of two ways: Either there is no thermal cost to lions in possessing a mane or a thermal cost to the mane does exist, but males compensate for the increased heat burden with increased heat loss. It seems intuitively obvious that a thick dark mane in a hot African savannah would be associated with a thermal cost as it may facilitate heat gain and impair heat loss. However, a darker and longer mane will not necessarily result in greater heat gain by the animal. Although a darker coloration may absorb more short‐wave radiation from the sun, the structure and density of hairs on an animals’ pelt influences the depth to which heat penetrates the pelt (Walsberg, 1983). In lions, the dark mane hairs are thicker than those of light mane hairs (West & Packer, 2002), which may act to increase the insulation of the pelt. A highly insulated pelt may create a “heat shield” that allows some of the radiant heat absorbed near the surface of a pelt to be dissipated back into the environment through conduction, convection, and radiation (Tattersall et al., 2012). For example, merino sheep in the unshaded desert planes of Australia had surface temperatures as high as 87°C, but a remarkable 45°C temperature gradient was sustained between the tip of the wool and the skin, by the 40‐mm‐thick pelage (Macfarlane, Morris, & Howard, 1956). In the case of a heat shield, pelt thickness and black (or dark) coloration can be beneficial (Tattersall et al., 2012). It is possible therefore that a long dark mane may allow males to remain in the sun for longer than for males with a short or light‐colored mane. Increased insulation by the mane may explain the weak but significant negative correlation we detected between mane length and 24‐hr mean *T*_b_, and why lions with dark manes had cooler 24‐hr mean *T*_b_ than those with medium‐colored manes. Nocturnal activity combined with shade‐seeking during the day by inactive lions (Schaller, 1972) also will further reduce the influence of mane color on radiant heat gain by lions.

Increased insulation in the neck and chest region is unlikely to impair the ability of male lions to lose heat at high ambient temperatures (similar to or above *T*_b_). When environmental temperatures exceed *T*_b_, as occurs frequently in savannah habitats occupied by lions, and in our study, heat can be dissipated only by evaporation. Domestic cats rely on panting for evaporative heat loss (Adams, Morgan, Hunter, & Holmes, 1970). They do not possess functional sweat glands over the body, and, contrary to a widely held view, sweat glands found on their paws (Munger & Brusilow, 1961) do not contribute to regulating heat balance (Adams et al., 1970). Like other cats, lions probably do not sweat across their fur and primarily rely on evaporative cooling during panting and licking of their fur, which is not impaired by the presence of the mane. When ambient temperatures are lower than *T*_b_, lions lose heat by rolling on their backs and exposing their thin‐skinned loins (Smith & Kok, 2006), which are not covered by the mane and may act as a thermal window. It is unlikely therefore that the mane impairs heat loss, particularly at high environmental temperatures when evaporative cooling is obligatory.

Although we have demonstrated that a darker and longer mane does not increase *T*_b_, it could be argued that males increase heat loss to compensate for a thermal cost of the mane. Such compensatory behavior would likely necessitate increased water intake, particularly for lions, like ours, in hot environments. The estimated required evaporation for thermoregulation decays exponentially in relation to body size (Schmidt‐Nielsen, 1997). Consequently, for a large sexually dimorphic mammal such as a lion, where males and females have different body masses (average female 126 kg, male 190 kg; [Skinner & Chimimba, 2005]), both sexes require about the same percentage of body mass (about 1.25% of body mass in the case of lions), for evaporation. Thus, males are expected to require about 50% more water for evaporative heat loss than are females.

Male lions did not drink more than females in terms of the total number of laps per drink episode, although male lions are larger than females, and their bigger mouths may allow them to ingest more water than females per lap; possibly making up the 50% extra water they require on account of their body size. However, we found that resident territorial male lions visited water points more than twice as frequently as did females or nomadic males, and this finding strongly suggests that water points are considered an important resource by resident (dominant) males. Dominant pride males are known to have longer, darker manes than nomadic males (West & Packer, 2002). Consequently, dominant males may visit water more frequently than nomadic males to increase water uptake to facilitate increased evaporative heat loss and compensate for a potentially greater heat load resulting from their large dark manes. In such cases, male lions with long dark manes would be signaling access to drinking water rather than greater tolerance of higher thermal loads, as was previously suggested (West & Packer, 2002). However, dominant male lions might frequent water points more than females for reasons other than a need to drink. For instance, water points are typically surrounded by an open piosphere (Washington‐Allen, van Niel, Ramsey, & West, 2004) that may constitute a good place for lions to roar because sound travels further. Water points may also provide definite locations in the landscape for territorial scent marking, which males might use more than females. Finally, water flux rates may also differ between males and females for reasons other than thermoregulation, such as higher rates of “spraying” by males during territorial scent marking, necessitating increased water uptake by males for reasons other than increased evaporative heat loss. Thus, the question of whether male lions increase evaporative heat loss to compensate for the thermal properties of the mane remains inconclusive, but it is clear that maned lions with access to water do not have higher *T*_b_ than females or than males with shorter manes. A comprehensive analysis of mane length and color collected at the population level from free‐living lion populations across different climates and degrees of access to drinking water would go some distance in understanding the thermal implications and ecological functions of the lions’ mane.

## Conflict of interest

None declared.
