# Supplementary material for: Getting to the core: Internal body temperatures help reveal the ecological function and thermal implications of the lions’ mane
Source: Ecol Evol. 2016 Dec 20;7(1):253–62. doi: 10.1002/ece3.2556 (PMC5214092; doi:10.1002/ece3.2556)
Supplement: Supplementary file 1 [file ECE3-7-253-s001.docx]

Table S1. Characteristics of the environment by season.

| **Environmental conditions** | **Season** | | | |
| --- | --- | --- | --- | --- |
| **Dry bulb temperature (°C)** | **Summer (n=56)** | **Autumn (n=92)** | **Winter (n=91)** | **Spring (n=91)** |
| 24 h mean | 26.2 ± 2.5^a^ | 22.1 ± 2.6^b^ | 18.7 ± 3.0^c^ | 25.5 ± 3.8^a^ |
| 24 h maximum | 33.5 ± 3.6^a^ | 32.5 ± 3.3^a,b^ | 31.2 ± 3.3^b^ | 35.8 ± 5.2^c^ |
| 24 h minimum | 20.9 ± 3.4^a^ | 14.5 ± 4.9^b^ | 8.2 ± 4.5^c^ | 16.7 ± 3.9^d^ |
| 24 h range | 12.6 ± 5.1^a^ | 18.0 ± 5.5^b^ | 23.0 ± 4.4^c^ | 19.1 ± 4.8^b^ |
| Absolute maximum | 42.8 | 39.1 | 40.5 | 46.1 |
| Absolute minimum | 14.7 | 5.0 | -0.5 | 7.4 |
| **Black globe temperature (°C)** |  |  |  |  |
| 24 h mean | 29.2 ± 3.4^a^ | 25.4 ± 3.1^b^ | 21.4 ± 3.7c | 28.1 ± 4.2^a^ |
| 24 h maximum | 47.1 ± 8.7^a^ | 48.3 ± 5.1^a^ | 42.8 ± 4.6b | 47.7 ± 7.2^a^ |
| 24 h minimum | 20.4 ± 4.0^a^ | 13.7 ± 5.3^b^ | 7.2 ± 5.3c | 15.8 ± 4.1^d^ |
| 24 h range | 26.7 ± 11.4^a^ | 34.7 ± 6.6^b,c^ | 35.6 ± 6.4b | 31.9 ± 7.0^c^ |
| Absolute maximum | 58.9 | 57.0 | 58.9 | 61.9 |
| Absolute minimum | 12.5 | 3.6 | -1.6 | 6.0 |
| **Other weather parameters** |  |  |  |  |
| 24 h wind speed (m s-1) | 1.0 ± 0.8^a^ | 0.6 ± 0.6^b^ | 0.9 ± 0.3^a^ | 1.9 ± 0.9^c^ |
| 24 h vapour pressure (kPa) | 2.2 ± 0.4^a^ | 1.7 ± 0.5^b^ | 0.9 ± 0.3^c^ | 1.3 ± 0.4^d^ |
| Mean monthly rainfall (mm) | 109 ± 49 | 56 ± 96 | 0 ± 0 | 13.8 ± 13.5 |

**Values with different superscript letters differed significantly, generalised linear model (p < 0.05). N denotes the number of days with data available in each season.*
