# Supplementary material for: Getting to the core: Internal body temperatures help reveal the ecological function and thermal implications of the lions’ mane
Source: Ecol Evol. 2016 Dec 20;7(1):253–62. doi: 10.1002/ece3.2556 (PMC5214092; doi:10.1002/ece3.2556)
Supplement: Supplementary file 2 [file ECE3-7-253-s002.docx]

Table S2. Summary of lion information and bio-loggers fitted.

| **Lion information** | | | **Bio-logger fitted** | | | | |
| --- | --- | --- | --- | --- | --- | --- | --- |
| **ID** | **Sex** | **Age** | | **temperature** | **accelerometer** | **acoustic-accelerometer** | **GPS collar** |
| Lion 1 | male | | adult |  | X |  | X |
| Lion 2 | male | | adult | X | X |  | X |
| Lion 3 | male | | sub-adult | X |  |  | X |
| Lion 4 | male | | adult |  |  |  | X |
| Lion 5 | male | | adult | X |  |  | X |
| Lion 6 | male | | adult | X | X |  | X |
| Lion 7 | male | | adult | X |  | X | X |
| Lion 8 | male | | adult | X | X | X | X |
| Lion 9 | male | | adult |  |  |  | X |
| Lion 10 | male | | adult | X | X |  | X |
| Lion 11 | male | | adult |  |  |  | X |
| Lion 12 | male | | adult | X | X |  | X |
| Lion 13 | male | | adult | X | X | X | X |
| Lion 14 | male | | adult | X | X | X | X |
| Lion 15 | male | | adult |  | X |  | X |
| Lion 16 | male | | adult | X | X | X | X |
| Lion 17 | male | | adult | X |  |  | X |
| Lioness 1 | female | | adult |  |  |  | X |
| Lioness 2 | female | | adult |  |  |  | X |
| Lioness 3 | female | | adult |  |  |  | X |
| Lioness 4 | female | | adult | X |  | X | X |
| Lioness 5 | female | | adult | X |  |  | X |
| Lioness 6 | female | | adult |  |  |  | X |
| Lioness 7 | female | | adult | X | X | X | X |
| Lioness 8 | female | | adult |  |  |  | X |
| Lioness 9 | female | | adult |  | X |  | X |
| Lioness 10 | female | | adult |  |  |  | X |
| Lioness 11 | female | | adult | X |  |  | X |
| Lioness 12 | female | | adult | X |  |  | X |
| Lioness 13 | female | | adult |  |  |  | X |
| Lioness 14 | female | | adult | X | X | X | X |
