# Supplementary material for: Getting to the core: Internal body temperatures help reveal the ecological function and thermal implications of the lions’ mane
Source: Ecol Evol. 2016 Dec 20;7(1):253–62. doi: 10.1002/ece3.2556 (PMC5214092; doi:10.1002/ece3.2556)
Supplement: Supplementary file 3 [file ECE3-7-253-s003.docx]

Table S3. Summary of collar data used in analysis of frequency of visits to water points.

| **ID** | **Sex** | **Status** | **Year** | **Days of data** | **Visits to water** | **Visits per day** |
| --- | --- | --- | --- | --- | --- | --- |
| Lion 1 | male | resident | 2014 | 91 | 44 | 0.48 |
| Lion 2 | male | resident | 2011 | 91 | 65 | 0.71 |
| Lion 2 | male | resident | 2012 | 91 | 63 | 0.69 |
| Lion 2 | male | resident | 2013 | 91 | 58 | 0.64 |
| Lion 2 | male | nomadic | 2014 | 91 | 13 | 0.14 |
| Lion 3 | male | nomadic | 2014 | 91 | 30 | 0.33 |
| Lion 4 | male | resident | 2013 | 91 | 44 | 0.48 |
| Lion 5 | male | nomadic | 2013 | 91 | 17 | 0.19 |
| Lion 6 | male | nomadic | 2013 | 91 | 28 | 0.31 |
| Lion 7 | male | nomadic | 2014 | 91 | 19 | 0.21 |
| Lion 8 | male | nomadic | 2014 | 91 | 26 | 0.29 |
| Lion 9 | male | resident | 2014 | 91 | 44 | 0.48 |
| Lion 10 | male | nomadic | 2012 | 81 | 10 | 0.12 |
| Lion 10 | male | resident | 2013 | 91 | 38 | 0.42 |
| Lion 11 | male | resident | 2013 | 88 | 42 | 0.48 |
| Lion 12 | male | nomadic | 2013 | 85 | 12 | 0.14 |
| Lion 12 | male | nomadic | 2014 | 91 | 27 | 0.3 |
| Lion 13 | male | resident | 2013 | 91 | 63 | 0.69 |
| Lion 13 | male | resident | 2014 | 91 | 53 | 0.58 |
| Lioness 1 | female | nomadic | 2014 | 91 | 10 | 0.11 |
| Lioness 2 | female | nomadic | 2014 | 91 | 16 | 0.18 |
| Lioness 3 | female | resident | 2014 | 91 | 10 | 0.11 |
| Lioness 4 | female | nomadic | 2014 | 91 | 15 | 0.16 |
| Lioness 5 | female | resident | 2013 | 85 | 24 | 0.28 |
| Lioness 5 | female | resident | 2014 | 91 | 6 | 0.07 |
| Lioness 6 | female | resident | 2013 | 90 | 13 | 0.14 |
| Lioness 6 | female | resident | 2014 | 91 | 20 | 0.22 |
| Lioness 7 | female | resident | 2012 | 80 | 26 | 0.33 |
| Lioness 7 | female | resident | 2013 | 82 | 12 | 0.15 |
| Lioness 7 | female | resident | 2014 | 91 | 6 | 0.07 |
| Lioness 8 | female | resident | 2013 | 91 | 23 | 0.25 |
| Lioness 9 | female | resident | 2011 | 91 | 31 | 0.34 |
| Lioness 9 | female | resident | 2014 | 91 | 33 | 0.36 |
| Lioness 10 | female | resident | 2013 | 91 | 18 | 0.2 |
| Lioness 10 | female | resident | 2014 | 91 | 4 | 0.04 |
| Lioness 11 | female | resident | 2013 | 91 | 30 | 0.33 |
| Lioness 12 | female | resident | 2013 | 89 | 29 | 0.33 |
| Lioness 12 | female | resident | 2014 | 91 | 17 | 0.19 |
| Lioness 13 | female | nomadic | 2013 | 84 | 43 | 0.51 |
