# Supplementary material for: Getting to the core: Internal body temperatures help reveal the ecological function and thermal implications of the lions’ mane
Source: Ecol Evol. 2016 Dec 20;7(1):253–62. doi: 10.1002/ece3.2556 (PMC5214092; doi:10.1002/ece3.2556)
Supplement: Supplementary file 4 [file ECE3-7-253-s004.docx]

Table S4. Summary of model selection statistics for linear mixed effects analysis of the relationship between 24 h T_b_ and the explanatory variables sex and season. Models were ranked according to Akaike weights (wj) based on the Akaike Information Criterion for small samples (AICc). Included are the number of parameters (K), the log likelihood (LogLik) and the AICc differences (Δi).

| Dependent variable | Model | Rank | K | logLik | AICc | Δi | wj |
| --- | --- | --- | --- | --- | --- | --- | --- |
| 24 h maximum T_b_ | season | 1 | 6 | -1.68 | 16.83 | 0 | 0.48 |
|  | season + sex | 2 | 7 | -0.47 | 16.95 | 0.119 | 0.453 |
|  | sex | 3 | 4 | -6.66 | 22 | 5.171 | 0.036 |
|  | sex * season | 4 | 10 | 0.92 | 22.32 | 5.486 | 0.031 |
| 24 h mean T_b_ | season + sex | 1 | 7 | 17.96 | -19.92 | 0 | 0.743 |
|  | season | 2 | 6 | 14.96 | -16.45 | 3.471 | 0.131 |
|  | season * sex | 3 | 10 | 20.26 | -16.36 | 3.56 | 0.125 |
|  | sex | 4 | 4 | 3.95 | 0.77 | 20.694 | 0 |
